# Spontaneous resolution of nonimmune hydrops fetalis in a fetus with *TP63* gene mutation and *LZTR1* gene variants

**DOI:** 10.1002/ccr3.4624

**Published:** 2021-08-10

**Authors:** Yannick Hurni, Martina Marangoni, Giulia Garofalo, Marie Cassart, Lisa Tomasi, Isabelle Vandernoot, Guillaume Smits, Caroline Gounongbé

**Affiliations:** ^1^ Department of Fetal Medicine CHU Saint‐Pierre Brussels Belgium; ^2^ Center of Human Genetics Hôpital Erasme Université Libre de Bruxelles Brussels Belgium; ^3^ Department of Pediatrics CHU Saint‐Pierre Brussels Belgium; ^4^ Department of Radiology Hôpitaux Iris Sud and CHU Saint‐Pierre Brussels Belgium

**Keywords:** EEC syndrome, fetal medicine, hydrops fetalis, LZTR1 gene, TP63 gene

## Abstract

In cases of fetal hydrops, searching for an etiology is essential to evaluate the fetal prognosis and propose the most appropriate management.

## Graphical abstract

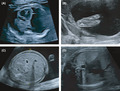

## 1 INTRODUCTION

We present a case of hydrops fetalis with spontaneous resolution and good postnatal cardiorespiratory outcomes in a fetus with *TP63* gene mutation linked to ectrodactyly, ectodermal dysplasia, cleft lip/palate syndrome 3, and two *LZTR1* gene variants of unclear phenotypic significance.

Hydrops fetalis is an excessive fluid accumulation within the fetal extravascular compartments and body cavities. It is defined by prenatal ultrasound as the presence of excessive fluid in more than one body cavity, with at least two of the following: subcutaneous edema, pleural effusion, pericardial effusion, and ascites. It is also frequently associated with thickened placenta and polyhydramnios.

Hydrops fetalis is caused by an imbalance in the regulation of fluid movements between the vascular and interstitial spaces, secondary to a wide variety of fetal, placental, and maternal conditions.^1^ The overall perinatal mortality and morbidity are high, and only a small number of cases show spontaneous regression or can be properly treated antenatally.^2^, ^3^ Here, we describe a case of hydrops fetalis with massive hydrothorax and generalized skin edema diagnosed at 21 weeks of gestation, with spontaneous resolution during the third trimester and good postnatal cardiorespiratory outcomes.

## 2 CASE REPORT

The parents gave the written consent of them and their child to participate in this study (approved by local Hôpital CHU Saint‐Pierre Ethical committee).

**FIGURE 1:**
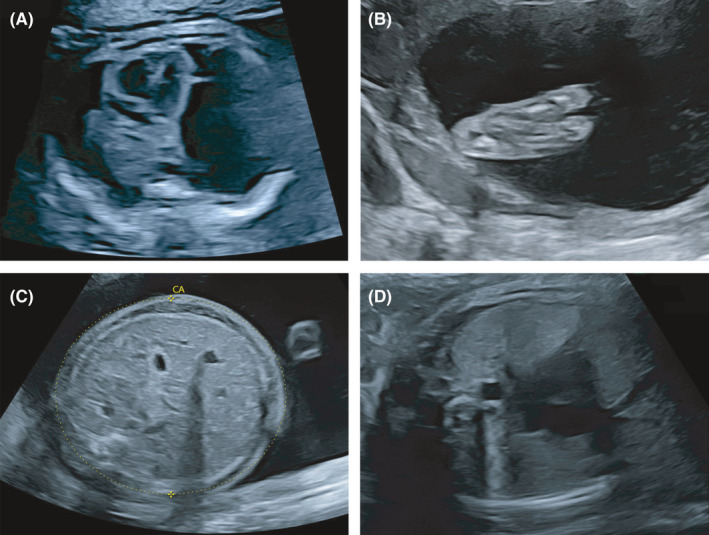
A, Ultrasound examination at 21 5/7 gestational weeks showing axial view of the fetal chest with bilateral hydrothorax and (B) ectrodactyly of the left foot. C, Ultrasound examination at 26 5/7 gestational weeks showing axial view of the fetal abdomen with periabdominal edema, no ascites and an abdominal circumference (CA) of 271.0 mm (>99th percentile). D, Ultrasound examination at 35 2/7 gestational weeks showing axial view of the fetal chest with hydrothorax resolution

A 32‐year‐old Congolese woman (gravida 3 and para 3) was referred to our service at approximately 21 weeks of gestation for ultrasound screening and pregnancy dating due to delayed follow‐up. The maternal medical anamnesis highlighted obesity (body mass index 36 kg/m^2^) and a chronic untreated arterial hypertension. She had a previous singleton pregnancy with term vaginal delivery complicated by postpartum preeclampsia and a bichorial‐biamniotic uncomplicated twin pregnancy with term vaginal delivery 3 and 2 years before, respectively. The couple is unrelated. The three children and the partner (a 35‐year‐old Congolese man) are in good health. The date of the last menstrual period was unknown, and the patient had only consulted once at approximately 19–20 weeks of gestation. The ultrasound examination performed at that time was reported to be normal. Two weeks later, the patient underwent an ultrasound screening in our unit. Pregnancy dating based on fetal biometry showed a fetus at 21 5/7 weeks of gestation. Ultrasonographic examination showed a hydrops fetalis with generalized edema (with minor impact on fetal biometry) and bilateral hydrothorax without ascites or pericardial effusion (Figure 1A). The left foot presented an ectrodactyly (Figure 1B), but the rest of the fetal morphology and the placenta seemed unremarkable. Amniotic liquid and feto‐maternal Dopplers were normal. Maternal blood tests and amniocentesis for prenatal genetic and microbiological analyses were performed. Immunological and infectious etiologies for the fetal hydrops were excluded.

**FIGURE 2:**
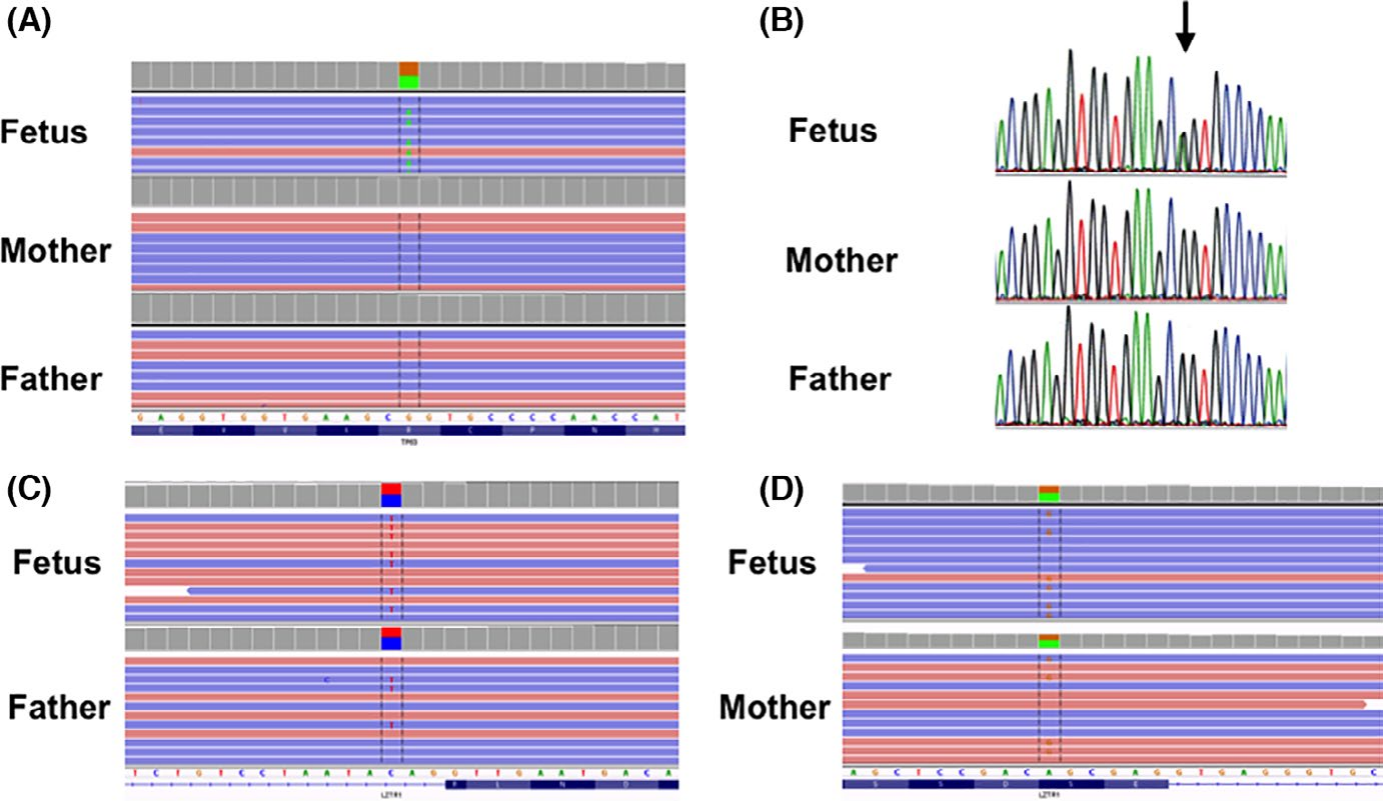
A, D*e novo* c.728G>A p.(Arg243Gln) variant (green block) in the TP63 gene visualized in Integrative Genomics Viewer (IGV); B, Validation of the c.728G>A p.(Arg243Gln) variant in the TP63 gene in the fetal DNA; C, IGV visualization of the c.594‐3C>T variant in the LZTR1 gene present in the fetus and his father (red block); D, IGV visualization of the c.988A>G p.(Ser330Gly) variant in the LZTR1 gene present in the fetus and his mother (orange block)

Quantitative fluorescence polymerase chain reaction for chromosomes 13, 18, 21, X, and Y was carried out to rapidly exclude the presence of the most common numerical chromosomal abnormalities. In addition, as array comparative genomic hybridization showed no clinical significant chromosomal abnormalities, clinical exome sequencing was proposed. The analysis performed in trio (fetal and parental DNA samples) revealed a *de novo* heterozygous NM_003722.5: c.728G>A p.(Arg243Gln) variant in the *TP63* gene in the fetal DNA extracted from uncultured cells of the amniotic fluid (Figure 2A), and this result was confirmed by Sanger sequencing (Figure 2B). In addition, two variants at a compound heterozygote state in the *LZTR1* gene were found: NM_006767.4: c.594‐3C>T (paternal origin) and c.988A>G p.(Ser330Gly) (maternal origin) (Figure 2C and 2D). As a trio‐based approach was performed, the LZTR1 variants were not validated by Sanger sequencing. No other interesting candidate variants were found after the *in silico* gene panel “Hydrops fetalis” analysis available on Genomics England PanelApp and the pCES trio analysis. We classified the *TP63* gene variant as pathogenic (class V) for the ectrodactyly, ectodermal dysplasia, cleft lip/palate syndrome 3 (EEC3), and the *LZTR1* gene variants as variants of unknown significance (class III), according to the international guidelines.^4^ After the genetic counseling, the parents decided to continue with the pregnancy. No fetal intervention has been proposed, and regular clinical and ultrasonographic examinations have been planned.

An ultrasound examination at 26 weeks of gestation revealed an increasing hydrops fetalis with massive hydrothorax and generalized edema (Figure 1C). We observed a polyhydramnios with an amniotic fluid index (AFI) of 29 cm, normal Doppler measurements, and no other abnormalities. The following ultrasound examinations at 33 and 35 weeks of gestation revealed a progressive spontaneous resolution of the fetal hydrothorax (Figure 1D) and a significant amniotic fluid volume reduction (AFI 6 cm). In addition, we observed a thickened placenta of 6 cm. Doppler measurements persisted normal, and fetal biometry revealed normal fetal growth on the 50th percentile.

The pregnancy continued uneventfully, and the mother was admitted at 40 weeks of gestation for spontaneous labor. She vaginally delivered a live male infant of 3810 g, with a body length of 48 cm and a head circumference of 35.5 cm. Apgar was 8/8/10, arterial pH was 7.34, base excess was −3.9 mEq/L, and lactate was 1.7 mmol/L. Due to temporary respiratory distress, the infant received 5 minutes of continuous positive airway pressure therapy with no other resuscitation maneuvers. Subsequently, he breathed spontaneously, and no longer needed any kind of respiratory support.

The first neonatal clinic examination revealed low‐set ears with pinna malformations, a thick and webbed neck, and almond‐shaped eyes with hypertelorism, but no cleft lip/palate or edema. The newborn presented a bell‐shaped skin outgrowth on the thorax between the nipples. Ectrodactyly was observed on the left foot, while the right hand presented a syndactyly of the third‐fourth fingers. While these malformations were not observed on the right foot and left hand, several nail implantations and shape abnormalities were observed on the four extremities. The rest of the examination appeared normal, and the neonate presented a regular neurological examination.

During the next few weeks, the infant underwent several examinations and evaluations, including cerebral, abdominal, and cardiac ultrasound; voiding urethrocystography; chest X‐ray; ophthalmologic examination; and auditory evoked potential measurements. They all appeared normal except for the observation of a bilateral renal pyelectasis of 10 and 8 mm.

**FIGURE 3:**
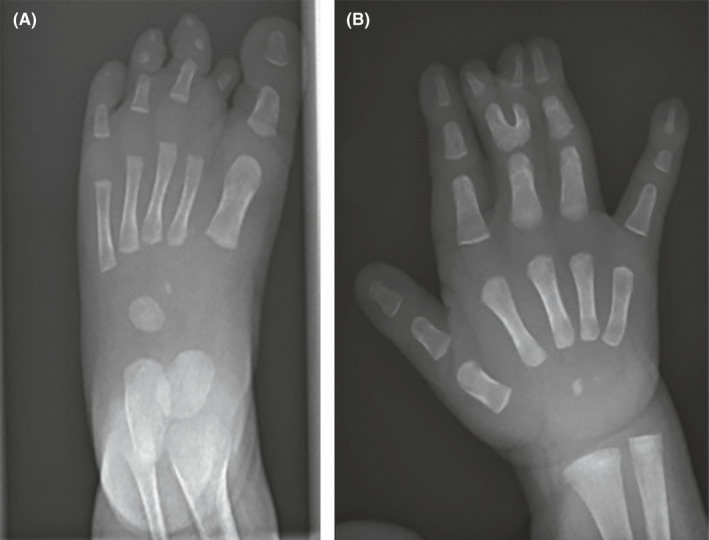
A, X‐rays at 10 months of age showing ectrodactyly on the left‐foot and (B) right‐hand syndactyly of the third‐fourth fingers

At 10 months of age, clinical and paraclinical examinations confirmed a phenotypic correlation with EEC3 **(**Figure 3), but no additional features suggesting for other genetic diseases have been observed. Examination of his parents and siblings revealed no similar or other relevant clinical findings. At this last control, the infant still presented no cardiorespiratory disorders, no neuro‐developmental delay, and general good outcomes.

## 3 DISCUSSION

Hydrops fetalis is an advanced stage of fetal decompensation due to an imbalance of fluid homeostasis.^1^ It is classically divided into immune (due to maternal hemolytic antibodies) and nonimmune (due to all other causes). Before routine immunization of rhesus‐negative patients, hydrops fetalis was most frequently caused by fetal anemia due to Rh hemolytic disease. Currently, most cases of hydrops fetalis are nonimmune in origin, whereas immune causes are only rarely observed. Due to difficulty in diagnosis and potential early spontaneous pregnancy termination, the incidence of hydrops fetalis varies considerably in different studies, ranging from 1.6 to 5.9 per 10,000 pregnancies.^5^, ^6^

Once a hydrops fetalis is diagnosed, clinicians should offer an attentive ultrasound examination searching for malformations, signs of fetal anemia, umbilical cord or placental irregularities, and cardiac functional abnormalities. In addition, genetics, serologic, and microbiologic examinations should be proposed. In a systematic review, Bellini et al. found that the most frequently encountered etiological factors were cardiovascular causes (21.4%), chromosomal imbalances (12.5%), and hematologic disorders (10.1%).^1^ Nevertheless, a consistent number of cases of hydrops fetalis remain unexplained (18.2%).^1^

In our case, the fetus developed severe hydrops fetalis at approximately 21 weeks of gestation, following unclear mechanisms. The genetic analyses on the amniotic liquid showed a normal molecular karyotype, a de novo heterozygous c.728G>A mutation in the *TP63* gene, and two compound heterozygous variants inherited from both parents in the *LZTR1* gene.

Anomalies of the *TP63* gene have been described as responsible for several phenotypes, including the EEC3 (OMIM: 604292), inherited in an autosomal dominant manner. The c.728G>A p.(Arg243Gln) variant has been already reported as pathogenic in several EEC3 patients in the literature.^7^, ^8^, ^9^, ^10^ Moreover, similar pathogenic variants were reported at the same amino acid position (c.727C>T p.(Arg243Trp); c.728G>T p.(Arg243Leu)).^7^, ^11^, ^12^, ^13^ Arg243 is localized in the DNA binding domain of the *TP63* protein, a domain where missense mutations are often described in EEC3 syndrome.^14^ The c.728G>A p.(Arg243Gln) variant is absent in the genome aggregation database (gnomAD). This variant is classified as pathogenic (class V). EEC3 is mainly characterized by limb anomalies (ectrodactyly with or without syndactyly in ~70% of all affected individuals), cleft lip/palate (~40%), and ectodermal defects.

Anomalies in the *LZTR1* gene have been associated with different phenotypes such as schwannomatosis type 2, (OMIM: 615670) inherited in an autosomal dominant manner with incomplete penetrance^15^ and Noonan syndrome,^16^ of both autosomal dominant (OMIM: 616564) and recessive (OMIM: 605275) transmission. The c.594‐3C>T variant is found with a frequency of 0.018% (50/282262 alleles) in gnomAD. This variant has not been reported in the literature, and it did not seem to have an impact on the splicing according to Human Splicing Finder (http://www.umd.be/HSF/HSF.shtml) and SpliceAI.^17^ However, a variant involving the same nucleotide (c.594–3C>G) and described as pathogenic has been reported at a heterozygote state in a patient presenting schwannomatosis type 2.^15^ The c.988A>G p.(Ser330Gly) variant was found with a frequency of 0.0019% (3/154198 alleles) in gnomAD, and it has not been reported in the literature. One variant involving the same amino acid residue (c.989G>T p.(Ser330Ile)) has been described as a variant of unknown significance in the Leiden Open Variation database (www.lovd.nl). Two *in silico* tools (MutationTaster, LRT) out of the six evaluated predicted a deleterious effect on protein function. This variant was localized at the end of the fifth Kelch motif (Uniprot Q8N653^18^).

In our case, the mutation in the *TP63* gene could explain the phenotype suggesting for EEC3, but we were unable to find any clear correlation with the hydrops fetalis. To our knowledge, only one case of EEC3 associated with hydrops fetalis has been reported, with the fetus presenting a large nephrogenic cyst with consequent renal dysplasia potentially causing the hydrops.^19^ *LZTR1* gene mutations have been associated with hydrops fetalis in a few cases,^20^, ^21^, ^22^ and all associated with Noonan syndrome and cardiac abnormalities. *LZTR1* gene mutations in Noonan syndrome seem to be linked to severe cardiac phenotypes, eventually associated with cardiogenic hydrops fetalis and prenatal death.^20^, ^21^ In contrast to the reported cases, our patient does not meet the diagnostic criteria for Noonan syndrome, and he presents no cardiac abnormalities. In addition, he showed prenatal hydrops resolution, perinatal survival, and good 10‐month cardiorespiratory outcomes.

Hydrops fetalis seems to resolve spontaneously in approximately 10% of cases and partially resolves in 30%.^3^, ^23^ This seems to be more likely when it is associated with certain etiologies, such as parvovirus infections.^24^ Nevertheless, only a few cases of idiopathic hydrops fetalis with prenatal resolution have been described^2^, ^25^, ^26^ and none in association with *LZTR1* gene mutations.^20^, ^21^, ^22^

The short‐ and long‐term prognoses for hydrops fetalis seem to be associated with the type, extent, and behavior of the accumulated liquids, the underlying causes, the associated malformations, the gestational age at presentation, the time to delivery, and eventual intrauterine interventions.^27^, ^28^ The overall perinatal survival rate appears to be between 25% and 55%, with the worst prognoses in cases of cystic hygroma, severe fetal edema, inborn error of metabolism, structure abnormalities, abnormal karyotype, and hydrops fetalis appearance at an early gestational age.^3^, ^27^, ^29^, ^30^, ^31^ Conversely, antenatal hydrops fetalis resolution, spontaneous or following an intrauterine treatment, and term delivery seem to be positive predictors for short‐ and long‐term survival.^3^, ^32^ The overall outcome for live‐born infants is poor, with the first‐year survival rate at approximately 50%.^5^, ^6^, ^27^, ^30^, ^32^

In our case, even if the hydrothorax was present between at least 21 and 26 gestational weeks, the newborn showed no respiratory complications and good perinatal outcomes. At the last medical control at 10 months of age, the infant still presented no cardiorespiratory disorders.

## 4 CONCLUSION

To our knowledge, this is the first reported case of hydrops fetalis associated with *TP63* gene mutation and *LZTR1* gene variants, presenting spontaneous resolution and good outcomes. We outline the importance of searching for an etiology in cases of hydrops fetalis in order to propose the most appropriate management. Considering the high number of unexplained cases with no clear prognoses, this case adds valuable information for clinicians.

## CONFLICT OF INTEREST

None declared.

## AUTHOR CONTRIBUTIONS

YH and MM: wrote the article. LT: examined the patient. MM, IV and GS: analyzed the genetics data. MC, GG, and CG: served as the author of the antenatal ultrasound examinations. MC, GS, and CG: coordinated the final version of the manuscript.

## Data Availability

Data available on request from the authors.
